# Functional evaluation for patients with lower extremity sarcoma: application of the Chinese version of Musculoskeletal Tumor Society scoring system

**DOI:** 10.1186/s12955-017-0685-x

**Published:** 2017-05-19

**Authors:** Leilei Xu, Xinhua Li, Zhou Wang, Jin Xiong, Shoufeng Wang

**Affiliations:** Department of Orthopedic Surgery, The Affiliated Drum Tower Hospital of Nanjing University Medical School, Zhongshan Road 321, Nanjing, 210008 China

**Keywords:** Extremity sarcoma, MSTS, Outcome, Reliability, Validity

## Abstract

**Background:**

The Musculoskeletal Tumor Society (MSTS) scoring system is a disease-specific instrument to determine the physical and mental health for patients with extremity sarcoma. This study aims to investigate the reliability and validity of the Chinese version of the MSTS, and to evaluate functional outcomes of the surgical treatment of lower extremity sarcoma using the Chinese MSTS.

**Methods:**

A cohort of 98 patients who had undergone surgery for lower extremity sarcoma were included. All the patients completed the clinical assessment with the Chinese MSTS and the Chinese Toronto Extremity Salvage Score (TESS). Assessment of psychometric properties was carried out through reliability and validity test. The reliability of Chinese MSTS was evaluated through test-retest analysis, inter-observer analysis and internal consistency. The inter-observer and test-retest reliability was analyzed with intra-class correlation coefficient (ICC). The internal consistency was evaluated by Cronbach’s α, with a value >0.70 considered acceptable. The discriminant validity was evaluated through comparison of the MSTS score between patients undergoing amputation surgeries and those undergoing limb-salvage surgeries. The construct validity was evaluated with the factor analysis.

**Results:**

The mean MSTS score was 21.5 ± 7.1. The ICC was 0.91 (95% confidence interval (CI) = 0.85–0.96) for the test-retest reliability and 0.90 (95% CI = 0.86–0.93) for the inter-observer analysis. The test for internal consistency showed a Cronbach’s α of 0.86 for the MSTS. Patients undergoing amputation surgery had remarkably lower MSTS score than patients undergoing limb-salvage surgeries (18.8 ± 5.4 vs. 23.5 ± 6.3, *p* = 0.005), which indicated a good discrinimant validity of the Chinese MSTS. The factor analysis indicated a 1-factor model with acceptable goodness of fit.

**Conclusions:**

The Chinese MSTS scoring system is a reliable and valid instrument with well-accepted psychometric properties. Through application of the Chinese MSTS, we demonstrated that patients receiving limb-salvage surgeries may have better functional outcome and QoL than those undergoing amputation surgeries.

**Electronic supplementary material:**

The online version of this article (doi:10.1186/s12955-017-0685-x) contains supplementary material, which is available to authorized users.

## Background

Sarcoma is a rare type of cancer that most frequently in the long bones and soft-tissue of the extremity [1, 2]. Previously, patients with extremity sarcoma were routinely treated by amputation surgery [3, 4]. Recently, with the development of adjuvant therapies in the form of radiation therapy and chemotherapy, more patients had the opportunity to undergo limb-salvage surgery [5]. Depending on the location and the progressive status of the sarcoma, amputation surgery may still be necessary, despite at the cost of compromised body image [3]. By contrast, limb-salvage surgery may result in long period of hospitalization and not optimally functioning outcome [6, 7]. Other disadvantages of limb-salvage surgery include the increased risk of infections and breakages of the prosthesis [6, 7]. It is noteworthy that patients with sarcoma may have a significant heterogeneity regarding individual factors, such as age, financial capacity and nature of the tumor. Therefore, it is very important to apply the most suitable treatment for each patient. In this perspective, it is necessary to develop a disease-specific instrument for patients with extremity sarcoma to determine their perceived physical and mental health.

Currently, several disease-specific instruments have been used to evaluate the functional outcome of patients with extremity tumors, such as Musculoskeletal Tumor Society (MSTS) rating scale [8, 9] and the Toronto Extremity Salvage Score (TESS) [10–13]. The MSTS Rating Scale is a widely used functional instrument which was developed in 1983 and later modified by the MSTS in 1993 [14]. The original version was written in English and has only been translated and validated in Brazilian and Japanese population [8, 9]. It is composed of six items, including pain, function, emotional acceptance, use of any external support, walking ability, and gait alteration. Each item was rated in a scale of 0 to 5. The total score ranges from 0 to 30, with higher scores indicating better function. The TESS questionnaire is a self-administered questionnaire evaluating functional difficulties. The TESS questionnaire of lower extremity was comprised of 29 questions rated on 5-point scale, including “impossible to do,” “extremely difficult,” “moderately difficult,” “a little bit difficult,” and “not at all difficult”. It has been cross-culturally adapted in the Chinese population, yielding good reliability and construct validity [13]. To the best of our knowledge, the validation of the Chinese version of the MSTS scoring system has not yet been reported. Therefore, in this study we applied the Chinese version of MSTS to a cohort of patients with lower extremity sarcoma. The objectives were to investigate the reliability and validity of the Chinese MSTS scoring system, and to evaluate functional outcomes of the surgical treatment of lower extremity sarcoma with the Chinese MSTS.

## Methods

### Subjects

The current study was approved by the local Institutional Review Board. Patients who underwent surgical treatment in our center between March 2008 and November 2015 were evaluated for the eligibility to be included in our study. The following inclusion criteria were used: 1. aged >18 years; 2. diagnosed as bone or soft-tissue sarcoma in the lower extremity; 3. with a minimum of 1 year follow-up after surgery; 4. without local recurrence or distant metastasis; 5. speaking mandarin during daily life. Finally, a cohort of 98 patients including 59 male and 39 female were included in the study. All the patients have received and completed neo-adjuvant chemotherapy for sarcoma in our center. The assessment was performed at the follow-up visit of the patients. A senior orthopedic surgeon (W.S.) administered the instrument to the patients. The MSTS score was calculated by two orthopedic surgeons independently (S.W and W.S.). No patient refused to complete the instrument, and there was no missing item. Patients’ medical records were used to collect the following demographic data, such as age, sex, tumor location, histological type of the tumor, type of surgical interventions and period of follow-up. Informed consents were obtained from all the patients.

### Translation of the Chinese MSTS

The protocol of translation of the instrument was performed as previously reported [13]. Briefly, the forward translation to Chinese was carried out independently by three bilingual translators. One of them speaks English as the mother language, and the other two are both orthopedic surgeons speaking Chinese (X.L. and W.Z.). The translations were then combined into one single version after a round-table discussion. The back translation to English was performed by two bilingual translators speaking English as mother language. An expert committee comprised of these five translators and two senior orthopedic surgeons (S.W and W.S.) reviewed all versions of the translations, and assessed conceptual equivalence of all items and discussed discrepancies. The pre-final version of the Chinese MSTS was created following a consensus within the expert committee. No modification was made regarding adaptation of the items since the original English version can well fit the Chinese lifestyle. Sixty healthy volunteers aged more than 18 years were recruited to test the pre-final Chinese version of the MSTS. All the items could be well completed by the volunteers. After this process, the final version of the Chinese version of the MSTS was created.

### Assessment of psychometric properties

The reliability was evaluated through test-retest analysis, inter-observer analysis and internal consistency. For the test-retest analysis, all the patients were asked to respond to the MSTS questionnaire at their follow-up visit after surgery and at 1 week after the visit. The data were collected by two independent orthopedic surgeons who were not involved in the treatment of the patients. The inter-observer and test-retest reliability was analyzed with intra-class correlation coefficient. The internal consistency was evaluated by Cronbach’s α, with a value >0.70 considered acceptable [8, 15]. In addition, the floor and ceiling effects were evaluated for the total score of the MSTS, which were considered positive if more than 15% of the subjects had a score of 0 or 30 [16].

For validity test, we evaluated the discriminant validity and concurrent validity. Based on the clinical judgment, we hypothesized that patients undergoing amputation surgery may have lower function scores than those receiving limb-salvage surgeries. We therefore compared the MSTS score between patients undergoing amputation surgeries and those undergoing limb-salvage surgeries. To examine the concurrent validity, patients were asked to complete both the Chinese version of the MSTS and the Chinese version of the TESS that has been previously validated in Chinese population. The total score of the TESS was converted by transforming the results to a range of 0 to 100, with higher scores indicating better function. Correlation between the total score of the MSTS and the TESS was analyzed by Pearson correlation analysis.

### Exploratory and confirmatory factor analysis

SPSS was used to perform the exploratory factor analysis (EFA) with varimax rotation to determine the latent factor structure. The varimax rotation was used to dimensionally separate the data cluster, which presented a spatial relationship among the factors. The Scree plot was used to determine the factor loading for each item. Variables with a factor loading of more than 0.4 were considered to be representative of the construct [8]. To further examine the latent factor structure, Akaike information criterion (AIC) network was used to determine the degree of correlation among the variables [8]. To verify the factor model, a confirmatory factor analysis (CFA) was performed with AOMS version 20 (IBM Corporation, 2011). The goodness-of-fit for the factor structure was evaluated using root mean square error of approximation (RMSEA), the comparative fit index (CFI), the goodness-of-fit index (GFI) and the non-normed fit index (NNFI) [9]. A model could be considered as a good fit when GFI, CFI, and NNFI were more than 0.9 and RMSEA was less than 0.06 [9].

### Statistical analysis

SPSS for Windows version 19.0 (SPSS, Chicago, IL, USA) was used for statistical analyses. Descriptive demographic data and scores were summarized as mean values ± standard deviation. The student t test was used to compare the MSTS score between male and female, and between patients aged ≥50 and those aged <50 years. *P* < 0.05 was considered statistically significant.

## Results

The Chinese MSTS and the Chinese TESS were completed successfully by all subjects. Table 1 summarized demographic data of the patients. The mean age was 50.8 ± 13.4 years (range, 18–61 years). The most common histological types were osteosarcoma (*n* = 31). The most frequent tumor location was in the thigh (*n* = 30). Amputation surgery was performed in 17 (17.3%) patients. 48 (48.9%) patients underwent complete resection of the tumor and reconstruction with prosthesis. The mean period of follow-up was 2.5 ± 1.4 years (range, 1–5 years). The mean MSTS score was 21.5 ± 7.1 (range, 6–30).

**Table 1:** Baseline characteristics of the patients

Characteristic	Patients (*n* = 98)
Age (years)	50.8 ± 13.4
Gender	
Male	59
Female	39
Time of follow-up (years)	2.5 ± 1.4
Tumor location	
Thigh	30
Lower leg	26
Pelvis/Hip	19
Knee	9
Ankle/Foot	14
Histological type	
Osteosarcoma	31
Chondrosarcoma	16
Ewing’s sarcoma	9
Liposarcoma	10
Undifferentiated pleomorphic sarcoma	7
Myxofibrosarcoma	4
Fibrosarcoma	4
Leiomyosarcoma	3
Epithelioid	2
Others	12
Type of surgery	
Amputation	17
Resection only	11
Resection + prosthesis	48
Resection + biological reconstruction	22

Assessment of psychometric properties of the Chinese MSTS was summarized in Table 2. The ICC for the test-retest reliability was 0.91 (95% confidence interval (CI) = 0.85–0.96). The ICC for the inter-observer reliability were 0.90 (95% CI = 0.86–0.93). The test for internal consistency showed a Cronbach’s α of 0.86 for the MSTS. The incidence of ceiling effect of the MSTS was 7.14% (7/98) and there was no case with floor effect. Concurrent validity analysis indicated significantly high correlation between the Chinese MSTS and the Chinese TESS (*r* = 0.745, *p* < 0.05). Patients undergoing amputation surgery had remarkably lower MSTS score than patients undergoing limb-salvage surgeries (18.8 ± 5.4 vs. 23.5 ± 6.3, *p* = 0.005). No significant difference of the MSTS score was found between male and female (21.1 ± 7.1 vs. 21.4 ± 7.7, *p* = 0.67), or between patients aged more than 50 yrs. and those aged less than 50 yrs. (21.6 ± 6.9 vs. 20.8 ± 7.3, *p* = 0.58).

**Table 2:** Assessment of psychometric properties of the MSTS and the TESS

	Minimum	Maximum	Mean	SD	Percent floor	Percent ceiling	ICC	95% CI
Test								
MSTS	6	30	21.5	7.1	0	7.14%	0.90^a^	0.86–0.93^a^
TESS	32	100	76.4	14.3	0	6.12%		
Retest								
MSTS	6	30	21.8	6.8	0	7.14%	0.91^b^	0.85–0.96^b^
TESS	33	100	77.3	15.2	0	6.12%	0.88^b^	0.83–0.93^b^

*MSTS* indicates Musculoskeletal Tumor Society; *TESS* indicates Toronto Extremity Salvage Score; *SD* indicates standard deviation; *ICC* indicates intraclass correlation coefficient; *CI* indicates confidential interval

^a^ICC for the inter-observer reliability; ^b^ICC for test-retest reliability

**Fig. 1:**
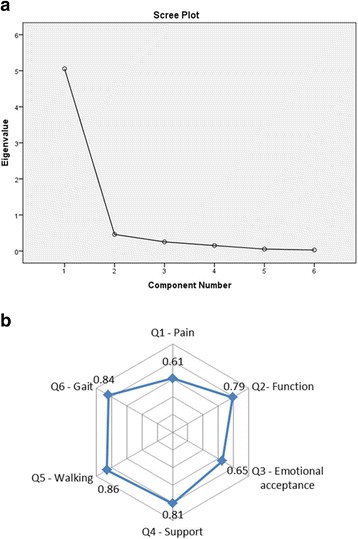
The factor structure analysis of the Chinese MSTS. **a** Scree plot with eigenvalues of the MSTS indicating 1-factor model with well-accepted fitness. **b** Factor loadings value for the MSTS shows that Walking had the highest value of 0.86

As shown in Fig. 1, the Scree plot indicated that the appropriate number of the factor was one. All six items had sufficient factor loading values, of which the item “walking” had the highest score of 0.86 (Fig. 1). The AIC network showed that “walking” and “gait” played a central role in the model. The CFA analysis showed an overall GFI of 0.93 and a RMSEA of 0.045. The CFI and the NNFI were 0.91 and 0.92, respectively.

## Discussion

The original MSTS questionnaire was a well-accepted rating system that facilitated valid evaluation of functional results after surgery of extremity sarcoma [14]. The advantage of this system lies in that it can be easily used in clinical practice and has acquired wide acceptance after extensive modifications and field trial. For the first time, we successfully translated and validated the Chinese version of the MSTS. During the translation process, we encountered no linguistic or cultural discrepancy. Our study demonstrated that the Chinese version has sufficient reliability and adequate construct validity as indicated by factor analysis and the AIC network.

Prior to this study, the original version MSTS has been validated in the Japanese population and the Brazilian population [8, 9]. Through psychometric analysis, these two versions of the MSTS scoring system were both confirmed to have sufficient reliability and validity. The ICCs for test-retest analysis were 0.92 in both the Japanese population and the Brazilian population [8, 9]. The ICC for the inter-observer analysis was 0.98 in the Brazilian population [9]. Comparably, in our study the ICCs for test-retest analysis and inter-observer analysis were 0.93 and 0.90, which demonstrated a higher level of reliability for Chinese MSTS. Moreover, we found a high internal consistency of Chinese MSTS as indicated by Cronbach’s α value of 0.86, which was similar to with a value of 0.87 for the Japanese version and a value of 0.84 for the Brazilian version [8, 9]. For the validity analysis, we investigated the correlation between Chinese MSTS and TESS that has been previously validated in Chinese population. Significantly high correlation coefficient indicated good concurrent validity of the Chinese MSTS. Using factor structure analysis we confirmed that the factor structure of the Chinese MSTS is acceptable. Through the different fit statistics, the 1-factor model was found reliable and trustworthy. Comparably, Iwata et al. [8] and Rebolledo et al. [9] both reported that one-factor model could produce the best fitness. Among the six factors of the model, we observed that “walking” had the highest loading values and played a central role in the net, which was similar to the finding of Iwata et al. [8]. Taken together, it appeared that the Chinese MSTS questionnaire could maintain the psychometric properties of the original version.

Along with innovative surgical procedures of lower extremity sarcoma, there has been a growing need to evaluate the functional outcomes and quality of life (QoL). QoL is an independent predictor of survival and response to therapy particularly among patients with cancer [17, 18]. Despite the intuitive speculation that patients with limb-salvage procedures should have better function and QoL than those receiving amputation, controversy still existed regarding the differences in outcomes for these patients [19–21]. Davis et al. [20] compared 12 patients undergoing amputation with 24 patients treated by limb-sparing surgery and reported no remarkable different in terms of the TESS score. By contrast, in a prospective multi-center study including 91 patients with lower-extremity bone sarcoma, Ginsberg et al. [19] observed that limb-sparing surgery can result in significantly higher TESS and MSTS score than amputation surgery. In this study, we confirmed that patients receiving amputation had lower MSTS score than patients undergoing limb-salvage surgery. In addition, we found that gender and age were not factors related to the postoperative MSTS score. On the basis of the Chinese MSTS, we were able to obtain highly consistent and reliable data supporting that limb-salvage surgery can achieve excellent functional long-term outcomes for patients with lower extremity sarcoma.

Two limitations still exist in this study. First, the current findings can be generalized only to adults with well-controlled sarcoma in remote phase after surgery. Whether the Chinese MSTS can be used to evaluate the functional outcome of patients with other types of lower extremity tumors needs to be further addressed. Second, the two instruments compared in our study were from different informants, as the TESS was self-reported while the MSTS was physician-reported. In future study, more general instruments should be included to demonstrate the validity of the Chinese MSTS.

## Conclusion

Chinese MSTS scoring system is a reliable and valid instrument with well-accepted psychometric properties. Through application of the Chinese MSTS, we demonstrated that patients receiving limb-salvage surgeries may have better functional outcome and QoL than those undergoing amputation surgeries.

## Additional file

Additional file 1: MSTS questionnaire in English version. (DOCX 17 kb)

## Abbreviations

CFA: A confirmatory factor analysis
EFA: Exploratory factor analysis
ICC: Intra-class correlation coefficient
MSTS: Musculoskeletal Tumor Society
QoL: Quality of life
TESS: Toronto Extremity Salvage Score
