# Supplementary material for: Functional evaluation for patients with lower extremity sarcoma: application of the Chinese version of Musculoskeletal Tumor Society scoring system
Source: Health Qual Life Outcomes. 2017 May 19;15:107. doi: 10.1186/s12955-017-0685-x (PMC5438502; doi:10.1186/s12955-017-0685-x)
Supplement: Additional file 1: — MSTS questionnaire in English version. (DOCX 17 kb) [file 12955_2017_685_MOESM1_ESM.docx]

**1. The items of the MSTS**

|  | **PAIN** | **FUNCTION** | **EMOTIONAL ACCEPTANCE** | **SUPPORT** | **WALKING** | **GAIT** |
| --- | --- | --- | --- | --- | --- | --- |
| 5 | None | No Restriction | Enthused | None | Unlimited | Normal |
| 4 |  |  | Intermediate |  |  |  |
| 3 | Modest | Recreational Restriction | Satisfied | Brace | Limited | Minor Cosmetic |
| 2 |  |  | Intermediate |  |  |  |
| 1 | Moderate | Partial Disability | Accepts | 1 Cane Crutch | Inside Only | Major Cosmetic Minor HCAP |
| 0 | Severe | Total Disability | Dislikes | 2 Cane Crutch | Unable Unaided | Major HCAP |

**2. The items of the TESS for lower extremity**

1. kneeling
2. rising from kneeling
3. gardening
4. performing heavy household duties
5. walking up and down hills
6. performing leisure activities
7. walking upstairs
8. bending
9. getting in and out of a bathtub
10. walking downstairs
11. getting in and out of a car
12. working usual number of hours
13. walking outdoors
14. putting on socks
15. performing work duties
16. shopping
17. putting on shoes
18. participating in sexual activities
19. walking indoors
20. putting on pants
21. preparing meals
22. showering
23. standing
24. sitting
25. performing light household duties
26. participating in social activities
27. driving
28. rising from a chair
29. getting in and out of bed

**Scoring**

1, “impossible to do,”; 2, “extremely difficult,”; 3, “moderately difficult,”; 4, “a little bit difficult,”; 5, “not at all difficult”.
